# Progression of cardiovascular autonomic neuropathy and cardiovascular disease in type 2 diabetes

**DOI:** 10.1186/s12933-018-0752-6

**Published:** 2018-08-02

**Authors:** Jae-Seung Yun, Yong-Moon Park, Seon-Ah Cha, Yu-Bae Ahn, Seung-Hyun Ko

**Affiliations:** 1 Division of Endocrinology and Metabolism, Department of Internal Medicine, St. Vincent’s Hospital, College of Medicine, The Catholic University of Korea, Ji-dong, Paldal-gu, Suwon, 16247 South Korea; 2 Epidemiology Branch, National Institute of Environmental Health Sciences, National Institutes of Health, Research Triangle Park, Durham, NC USA

**Keywords:** Cardiovascular autonomic neuropathy, Cardiovascular disease, Type 2 diabetes

## Abstract

**Background:**

To examine whether the progression rate of cardiovascular autonomic neuropathy (CAN) stage is an independent predictive factor for cardiovascular disease (CVD) in type 2 diabetes.

**Methods:**

Standardized cardiovascular autonomic reflex tests (CARTs) using traditional Ewing method were performed at baseline. The follow-up CARTs was recommended once every two years. We estimated the primary CVD endpoint, defined as coronary artery disease and ischemic stroke. The association between the progression rate of CAN stage and CVD was examined using time-dependent Cox proportional hazard models.

**Results:**

At baseline, 578 patients completed follow-up CARTs; the cohort comprised 329 women (56.9%) with a mean age of 58.3 ± 10.3 years and a mean diabetes duration of 10.1 ± 6.2 years. One hundred and seventy-six patients (30.4%) developed CAN progression between baseline and follow-up CARTs. In the multivariable Cox proportional hazards regression analysis, patients with CAN progression demonstrated a 3.32 times higher risk (95% confidence interval, CI 1.81–6.14, *P* < 0.001) of CVD than those without CAN progression. Patients who experienced CAN progression from the normal to definite stage had the greatest risk of CVD compared to other patients (hazard ratio 4.91, 95% CI 2.05–11.77, P for trend = 0.001).

**Conclusions:**

CAN stage progression was associated with an increased risk of CVD in this type 2 diabetes cohort. Patients with rapid CAN progression had the greatest risk of CVD. Thus, regular screening and risk management of CAN progression is necessary to prevent CVD.

**Electronic supplementary material:**

The online version of this article (10.1186/s12933-018-0752-6) contains supplementary material, which is available to authorized users.

## Background

Cardiovascular autonomic neuropathy (CAN) is one of the most common and serious complications associated with diabetes and is defined as the impairment of the autonomic control of the cardiovascular system [1]. CAN is caused by damage to the autonomic nerve fibers that innervate the heart and blood vessels, leading to abnormal control of heart rate and cardiac performance [2]. Clinical manifestations of CAN are resting tachycardia, exercise intolerance, orthostatic hypotension, and silent myocardial infarction [3], which can affect the daily activities and quality of life of patients with diabetes and may lead to life-threatening outcomes [4]. Thus, CAN assessment is important for establishing a strategy for diabetes care and for predicting the prognosis of patients with diabetes.

CAN is a progressive disease entity [3, 5] that is considered to progress from a subclinical stage, characterized by abnormalities of heart rate variability, to a clinically apparent stage with diverse and disabling symptoms [5]. Risk factors associated with CAN include hyperglycemia, duration of diabetes, hypertension, dyslipidemia, and obesity in type 2 diabetes [6]. Cardiovascular autonomic reflex tests (CARTs) are the most commonly used methods for the diagnosis of CAN and can easily assess cardiovascular autonomic function based on heart rate response to deep breathing, Valsalva maneuver, and postural change [7, 8]. Generally, the presence of CAN is associated with poor outcomes among diabetes complications [1, 9–12].

Several previous studies suggested that CAN contributes to an increased risk of cardiovascular disease (CVD) and CVD-related mortality. Heart rate variability is a well-known predictive factor of silent myocardial infarction, recurrent CVD, and post-myocardial infarction mortality [13–15]. However, no study has evaluated the association between the progression of CAN and cardiovascular events among patients with type 2 diabetes. We hypothesized that a more rapid CAN progression is related to poorer outcomes of cardiovascular events. This study aimed to evaluate the association between the progression of CAN and CVD using standardized CARTs in patients with type 2 diabetes.

## Methods

### Participants

This prospective study was approved by the Ethics Committee of the Catholic Medical Center and was performed according to the Declaration of Helsinki. All participants provided signed written informed consent. From 2000 to 2008, 1073 patients with type 2 diabetes were included and underwent baseline CARTs at the University-affiliated Diabetes Center of St. Vincent’s Hospital in South Korea. Patients were excluded if they had a history of CVD and any form of severe diseases such as: severe infection, liver cirrhosis, malignancy, or heart failure. Patients with definite CAN (autonomic function test score ≥ 2) or arrhythmia (e.g., atrial fibrillation) were also excluded at the initial visit and follow-up, which is the second test period. The study design summary is shown in Additional file 1: Figure S1.

### CAN evaluation

CARTs were performed using the standardized Ewing method with the Monitor One nDX device (QMed Inc., Eatontown, NJ). CARTs included a test of heart rate variability, including the R–R response to paced breathing (expiration/inspiration ratio, E/I ratio), Valsalva maneuver, and postural change from lying to standing, as previously described [6]. Patients were asked to fast for 12 h before the autonomic function test and to avoid taking insulin, anti-depressants, neuroleptic agents, caffeine, nicotine, antihistamines, or sympatholytic drugs that could affect the results of the cardiovascular autonomic test [7]. E/I ratios below the age-related reference value, Valsalva ratios < 1.20, and posture ratios < 1.03 were considered abnormal [16]. Each of the ratios was calculated as normal (0) or abnormal (1), with a maximum total score of 3. A CAN stage score of 0 was defined as a normal autonomic function, whereas scores of 1 and 2 or more were defined as early CAN and definite CAN, respectively [6]. The follow-up CARTs was recommended once every two years unless there were any specific CAN-related symptoms within 1 year following baseline CARTs. CAN progression was defined as an increased score in the follow-up test compared with the score in the baseline test. The progression group was categorized into the following 4 subgroups based on the status at baseline and follow-up CAN stages: non-progression, normal to early stage, early to definite stage, and normal to definite stage.

### Data collection

At follow-up, second autonomic function test, patient height, weight, and blood pressure were measured. Hypertension was defined as systolic blood pressure ≥ 140 mmHg, diastolic blood pressure ≥ 90 mmHg, or any use of antihypertensive medications. All laboratory measurements were performed after a 12-h overnight fast. Fasting plasma glucose levels were measured using an automated enzymatic method, with HbA1c levels determined using high-performance liquid chromatography. Blood lipid concentrations of total cholesterol, triglycerides, HDL-cholesterol, and LDL-cholesterol levels were measured enzymatically using an automatic analyzer. HbA1c levels were measured at least once every 6 months, and lipid profiles were measured at least once each year to evaluate glycemic control. Mean HbA1c and mean lipid profiles were calculated as the average HbA1c and lipid profile levels during the period from baseline CARTs to follow-up CARTs. These were used to adjust the baseline glycemic status in this analysis (Additional file 1: Figure S1). Urinary albumin excretion was measured via immunoturbidimetry (Eiken, Tokyo, Japan) with 24-h urine collection at baseline. To determine renal function, patients’ serum creatinine and eGFR levels were assessed. Additionally, a kinetic picrate method (Jaffe method) was used to determine serum creatinine levels. The most recent Chronic Kidney Disease Epidemiology Collaboration equations were used to calculate patients’ eGFR and measure the serum creatinine level [17].

### Cardiovascular outcomes

Patients who underwent follow-up second CARTs received follow-up clinical care until December 2016 (Additional file 1: Figure S1). The primary endpoint was the development of the first cardiovascular event. CVD was defined as a diagnosis of coronary artery disease or stroke. Coronary artery disease included angina pectoris, myocardial infarction, or coronary revascularization (coronary bypass, surgery, or coronary angioplasty) [18]. Stroke history included a previous transient ischemic attack or cerebral infarction [19]. We verified the onset of CVD via interviews at every visit and by using medical records. A diagnosis of a cardiovascular event was confirmed by specialists, including a cardiologist, neurologist, and neurosurgeon. The cause and time of death were obtained from hospital records or by making regular telephone call checks when patients did not attend a follow-up.

### Statistical analysis

Baseline characteristics are presented as means and standard deviations or medians with interquartile ranges. Chi square tests were used to assess the differences in the proportion of categorical variables. Independent Student *t*-tests were used for evaluating differences between the means of 2 continuous variables. Kaplan–Meier plots were used to illustrate the cumulative incidence of the first cardiovascular event according to CAN progression. We used Cox proportional hazard regression analysis to assess the associations between outcomes and potential explanatory variables. Proportional hazards assumptions were examined using log-minus log-survival plots. There was no significant departure from proportionality in hazards over time. A time-dependent Cox proportional hazards regression model was used to identify associations between CAN and CVD, with CAN progression considered the time-dependent variable. Potential confounders were identified a priori based on literature reviews. The first model was the crude model; the second was the age and sex-adjusted model. The third model was analyzed after adjusting for the following risk factors: sex, age, duration of diabetes, presence of hypertension, body mass index (BMI), smoking, alcohol consumption, use of medications [insulin, ACE inhibitor, angiotensin II receptor blocker (ARB), aspirin, statin], mean HbA1c, standardized deviation of HbA1c levels, and mean LDL-cholesterol level between first assessment and follow-up CARTs period, urinary albumin excretion rate, and estimated GFR. We performed an additional analysis, which included the group with definite CAN at initial CARTs, in order to compare CVD risk between the group with definite CAN during initial CARTs and that with CAN progression. The associations between CAN progression and other variables were examined to determine the effect modification in this model. When *P* for an interaction was significant we performed stratified subgroup analysis. Statistical analyses were performed using SAS version 9.3 (SAS Institute Inc., Cary, NC, USA). *P* value, *P* for trend, and *P* for interaction were considered statistically significant at < 0.05.

## Results

In this study, 174 patients (30.1%) had CAN progression between baseline and follow-up CARTs. The mean duration between the two assessments was 2.3 years. The screening intervals between baseline and follow-up CARTs were not different between the CAN stage groups (Additional file 2: Table S1). Patients with CAN progression were older; had a longer duration of diabetes; used more insulin; and had a higher level of HbA1c, fasting plasma glucose, LDL-cholesterol, and urinary albumin excretion (Table 1). Of patients with CAN progression, progression from normal to early CAN was diagnosed in 79 patients (45.4%), progression from early to definite CAN in 65 (37.3%), and progression from normal to definite CAN in 30 (17.2%). The patients who progressed from the normal to definite CAN stage had a higher fasting plasma glucose level (9.4 ± 1.5 mmol/L vs. 8.4 ± 2.9 mmol/L, *P* < 0.001), mean HbA1c [9.2 ± 1.4% (77.4 ± 15.6 mmol/L) vs. 8.3 ± 1.5% (67.0 ± 16.0 mmol/L), *P* < 0.001), and mean LDL-cholesterol level (3.2 ± 0.6 mmol/L vs. 2.7 ± 0.8 mmol/L, *P* = 0.004) compared with other patients who did not progress from the normal to definite CAN stage. However, there were no differences in age, duration of diabetes, presence of hypertension, BMI, smoking, alcohol consumption, use of medications, estimated GFR, and albumin excretion rate between the subgroups (Table 2). Among patients who progressed from normal to definite CAN, 86.7% had a score of 2, 93.3% had an abnormal Valsalva ratio score, and 83.3% showed an abnormal posture ratio score. There was no difference in the interval duration of CARTs (from baseline to follow-up CARTs) between the subgroups (*P* = 0.126).

**Table 1:** Baseline parameters between the group with and without progression of cardiovascular autonomic neuropathy

	Total	CAN progression (−)	CAN progression (+)	*P*-value
	(N = 578)	(N = 404)	(N = 174)	
Women, n (%)	329 (56.9)	220 (54.5)	109 (62.6)	0.083
Age (years)	58.3 ± 10.3	57.1 ± 10.1	60.9 ± 10.3	< 0.001
Diabetes duration (years)	10.1 ± 6.2	9.1 ± 5.7	12.4 ± 6.7	< 0.001
Body mass index (kg/m^2^)	24.7 ± 3.2	24.8 ± 3.2	24.7 ± 3.4	0.710
Hypertension, n (%)	242 (43.8)	163 (42.2)	79 (47.6)	0.284
Smoking, n (%)	124 (21.5)	93 (23.0)	31 (17.8)	0.198
Alcohol, n (%)	138 (23.9)	105 (26.0)	33 (19.0)	0.087
Insulin, n (%)	141 (24.4)	87 (21.5)	54 (31.0)	0.020
ACE inhibitor/ARBs, n (%)	181 (31.3)	122 (30.2)	59 (33.9)	0.433
Calcium channel blocker, n (%)	101 (17.5)	63 (15.6)	38 (21.8)	0.090
Aspirin, n (%)	45 (7.8)	28 (6.9)	17 (9.8)	0.318
Statin, n (%)	65 (11.2)	44 (10.9)	21 (12.1)	0.789
FPG (mmol/L)	8.4 ± 2.9	8.3 ± 2.7	8.8 ± 3.3	0.053
eGFR (mL/min/1.73 m^2^)	85.6 ± 17.1	86.7 ± 16.1	83.3 ± 19.0	0.040
Mean HbA1c (%)	8.3 ± 1.5	8.1 ± 1.5	8.8 ± 1.6	< 0.001
SD HbA1c	1.1 ± 1.0	1.1 ± 1.0	1.0 ± 1.0	0.787
Mean HbA1c (mmol/L)	67.1 ± 16.7	65.1 ± 16.0	71.5 ± 17.6	< 0.001
Mean total cholesterol (mmol/L)	4.7 ± 0.9	4.7 ± 0.9	4.8 ± 0.9	0.078
Mean triglyceride (mmol/L)	1.7 ± 1.0	1.7 ± 1.0	1.7 ± 0.9	0.931
Mean HDL-cholesterol (mmol/L)	1.2 ± 0.3	1.2 ± 0.3	1.1 ± 0.3	0.204
Mean LDL-cholesterol (mmol/L)	2.8 ± 0.8	2.7 ± 0.8	2.9 ± 0.8	0.021
UAE (mg/day)	79.8 ± 298.9	48.1 ± 191.4	153.8 ± 453.3	0.004

Values are presented as number (%) or mean ± SD

*CAN* cardiovascular autonomic neuropathy, *ARB* angiotensin receptor blocker, *FPG* fasting plasma glucose, *eGFR* estimated glomerular filtration rate, *SD* standard deviation, *UAE* urinary albumin excretion

**Table 2:** Descriptive characteristics according to the progression status of cardiovascular autonomic neuropathy

	Non-progression	Normal → early	Early → definite	Normal → definite	*P*-value
	(N = 404)	(N = 79)	(N = 65)	(N = 30)	
Women, n (%)	220 (54.5)	49 (62.0)	41 (63.1)	19 (63.3)	0.341
Age (years)	57.1 ± 10.1	59.9 ± 10.0	62.0 ± 11.1	60.7 ± 8.8	< 0.001
Diabetes duration (years)	9.1 ± 5.7	11.4 ± 6.6	14.2 ± 7.1	11.5 ± 5.6	< 0.001
Body mass index (kg/m^2^)	24.8 ± 3.2	24.2 ± 3.4	24.7 ± 3.4	25.7 ± 2.9	0.518
Hypertension, n (%)	163 (42.2)	35 (46.7)	31 (50.0)	13 (44.8)	0.655
Smoking, n (%)	93 (23.0)	12 (15.2)	15 (23.1)	4 (13.3)	0.295
Alcohol, n (%)	105 (26.0)	18 (22.8)	13 (20.0)	2 (6.7)	0.091
Insulin, n (%)	87 (21.5)	14 (17.7)	30 (46.2)	10 (33.3)	< 0.001
ACE inhibitor/ARBs, n (%)	122 (30.2)	25 (31.6)	24 (36.9)	10 (33.3)	0.742
Calcium channel blocker, n (%)	63 (15.6)	20 (25.3)	13 (20.0)	5 (16.7)	0.198
Aspirin, n (%)	28 (6.9)	7 (8.9)	6 (9.2)	4 (13.3)	0.57
Statin, n (%)	44 (10.9)	9 (11.4)	8 (12.3)	4 (13.3)	0.968
FPG (mmol/L)	8.3 ± 2.7	8.4 ± 2.8	9.1 ± 4.3	9.4 ± 1.5	0.006
eGFR (mL/min/1.73 m^2^)	86.7 ± 16.1	84.3 ± 17.7	81.1 ± 20.6	85.2 ± 18.9	0.042
Mean HbA1c (%)	8.1 ± 1.5	8.2 ± 1.4	9.2 ± 1.6	9.2 ± 1.4	< 0.001
SD HbA1c	1.1 ± 1.0	0.7 ± 0.6	1.3 ± 1.3	1.2 ± 1.1	0.285
Mean HbA1c (mmol/L)	65.1 ± 16.0	64.8 ± 16.3	76.9 ± 17.3	77.4 ± 15.6	< 0.001
Mean total cholesterol (mmol/L)	4.7 ± 0.9	4.7 ± 0.9	4.7 ± 0.9	5.2 ± 0.8	0.009
Mean triglyceride (mmol/L)	1.7 ± 1.0	1.7 ± 0.8	1.6 ± 0.9	1.8 ± 1.1	0.971
Mean HDL-cholesterol (mmol/L)	1.2 ± 0.3	1.1 ± 0.3	1.1 ± 0.3	1.2 ± 0.3	0.445
Mean LDL-cholesterol (mmol/L)	2.7 ± 0.8	2.8 ± 0.8	2.8 ± 0.9	3.2 ± 0.6	0.003
UAE (mg/day)	48.1 ± 191.4	142.5 ± 481.0	182.7 ± 513.5	120.4 ± 137.9	0.001

Values are presented as number (%) or mean ± SD

*CAN* cardiovascular autonomic neuropathy, *ARB* angiotensin receptor blocker, *FPG* fasting plasma glucose, *eGFR* estimated glomerular filtration rate, *SD* standard deviation, *UAE* urinary albumin excretion

**Fig. 1:**
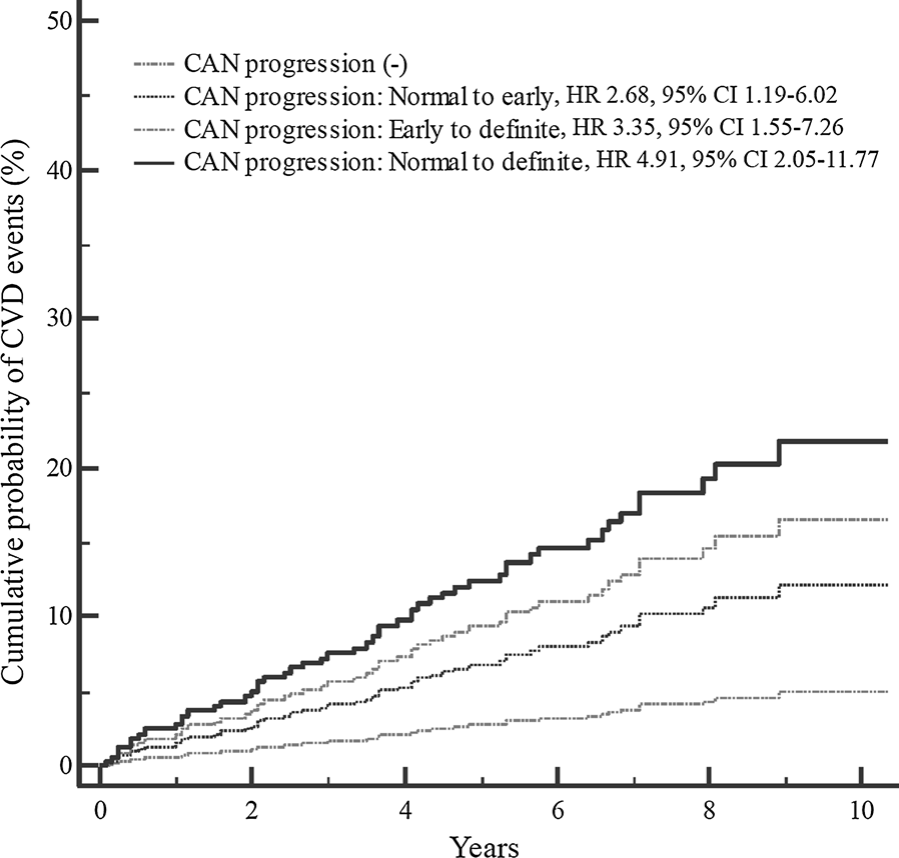
Cumulative probability of cardiovascular events according to the progression status of cardiovascular autonomic neuropathy. *CVD* cardiovascular disease, *CAN* cardiovascular autonomic neuropathy

The median time for follow-up was 7.3 years. During the study period, a CVD event occurred in 55 patients (9.3%). The overall incidence rate of a CVD event was 1.27 per 100 patient-years. Patients with CVD were older, had longer diabetes duration, used more insulin, and had a higher level of HbA1c at baseline, as well as lower eGFR levels (data not shown). The incidence of CVD increased in the group with CAN progression. This group demonstrated the highest rate of progression from normal to definite CAN. In the multivariate Cox proportional hazards regression analysis, patients with CAN progression had a 3.32 times higher risk of CVD than those without CAN progression. Patients who progressed from normal to definite CAN during the study period had a greatest risk of CVD than those of other patients following adjustment for potential confounders (Table 3, Fig. 1). We performed an additional analysis after including the group with definite CAN at baseline CARTs. The group with CAN progression (both early to definite and normal to definite) showed a higher risk for CVD compared to the group with maintained definite CAN (Additional file 2: Tables S2 and S3).

**Table 3:** Crude and multivariable Cox proportional hazard model for cardiovascular disease and sensitive analysis for cardiovascular disease after exclusion of the patients who developed cardiovascular disease within 2 years

	Crude HR (95% CI)	*P* value	Age and sex adjusted HR (95% CI)	*P* value	Fully adjusted HR (95% CI)	*P* value
*Overall risk of CVD*						
Non-progression of CAN	Reference		Reference		Reference	
Progression of CAN	4.31 (2.49–7.47)	< 0.001	3.68 (2.10–6.47)	< 0.001	3.32 (1.81–6.14)	< 0.001
Non-progression	Reference		Reference		Reference	
Normal to early	2.73 (1.28–5.81)	0.009	2.30 (1.07–4.94)	0.032	2.68 (1.19–6.02)	0.017
Early to definite	5.18 (2.63–10.20)	< 0.001	4.29 (2.12–8.66)	< 0.001	3.35 (1.55–7.26)	0.002
Normal to definite	6.99 (3.19–15.32)	< 0.001	6.58 (2.99–14.47)	< 0.001	4.91 (2.05–11.77)	< 0.001
P for trend	< 0.001		< 0.001		0.001	
*Subgroup analysis*						
Age < 60 years						
Non-progression of CAN	Reference		Reference		Reference	
Progression of CAN	8.04 (3.01–21.51)	< 0.001	8.29 (3.05–22.51)	< 0.001	5.49 (1.89–16.00)	0.001
Age ≥ 60 years						
Non-progression of CAN	Reference		Reference		Reference	
Progression of CAN	2.59 (1.33–5.03)	0.005	2.46 (1.25–4.81)	0.009	2.29 (1.10–4.77)	0.03
Diabetes duration < 10 years						
Non-progression of CAN	Reference		Reference		Reference	
Progression of CAN	5.37 (2.16–13.38)	< 0.001	4.40 (1.73–11.17)	0.002	5.34 (1.81–15.70)	0.002
Diabetes duration ≥ 10 years						
Non-progression of CAN	Reference		Reference		Reference	
Progression of CAN	3.20 (1.58–6.49)	0.001	3.02 (1.48–6.16)	0.002	2.67 (1.24–5.74)	0.012
BMI < 25 kg/m^2^						
Non-progression of CAN	Reference		Reference		Reference	
Progression of CAN	5.28 (2.43–11.47)	< 0.001	4.86 (2.22–10.64)	< 0.001	4.87 (2.00–11.90)	0.001
BMI ≥ 25 kg/m^2^						
Non-progression of CAN	Reference		Reference		Reference	
Progression of CAN	3.70 (1.66–8.26)	0.001	2.81 (1.22–6.48)	0.016	3.39 (1.38–8.37)	0.008
Mean HbA1c < 9.0%						
Non-progression of CAN	Reference		Reference		Reference	
Progression of CAN	3.47 (1.67–7.20)	0.001	2.88 (1.35–6.16)	0.006	3.16 (1.41–7.10)	0.005
Mean HbA1c < 9.0%						
Non-progression of CAN	Reference		Reference		Reference	
Progression of CAN	4.47 (1.86–10.72)	0.001	3.91 (1.61–0.95)	0.003	5.44 (2.01–14.72)	0.001
*Sensitive analysis*						
Non-progression of CAN	Reference		Reference		Reference	
Progression of CAN	3.41 (1.81–6.43)	< 0.001	2.94 (1.54–5.63)	0.001	2.85 (1.40–5.78)	0.004

*CVD* cardiovascular disease, *CAN* cardiovascular autonomic neuropathy

We analyzed the association between CAN progression and the risk of CVD within subgroups stratified by sex, age, diabetes duration, BMI, hypertension, HbA1c, and LDL-cholesterol. The association between CAN progression and CVD increased in participants aged < 60 years, with a diabetes duration < 10 years, a BMI < 25 kg/m^2^, and a mean HbA1c > 9% (*P* for interaction < 0.001). In contrast, sex, presence of hypertension, and mean LDL-cholesterol were not significantly associated with CAN progression and CVD (Table 3). The results of the sensitivity analyses after excluding patients who developed CVD within 2 years did not alter the significance of the association between CAN progression and CVD development (Table 3). During the follow-up period, 18 patients (3.1%) died, of which 5 (0.9%) died owing to CVD. The overall mortality rate was significantly higher in patients with CAN progression (6.9% vs. 1.5%, *P* < 0.001).

## Discussion

In this prospective study, we demonstrated a significant association between CAN progression and CVD risk in patients with type 2 diabetes. Notably, patients with rapid CAN progression had the highest risk of CVD.

### Risk factors of CAN progression

The progression and natural history of CAN are not completely understood. Findings from recent studies suggest some of the associated factors for CAN. Serum average and variability of glycated albumin level, which represent the short-term (2–3 weeks) glycemic status, are significantly associated with the presence of CAN [20]. Shimabukuro et al. demonstrated that short-term suppression of glycemic variability with α-glucosidase inhibitor can improve sympathetic nervous system activity and modify heart rate variability [21]. Jaiswal et al. found that the prevalence of CAN in a young diabetes cohort was comparable to that reported in the adult diabetes population and that elevated triglyceride levels were the modifiable factors associated with CAN in this group [22]. Another recent report suggested that apolipoprotein A-1 level can be a useful marker for the presence of CAN [23].

In this study, the cumulative incidence of CAN was approximately 30%, and 17.6% of patients in the progression group developed definite CAN over 2.3 years. The factors associated with CAN progression were age, diabetes duration, use of insulin, and increased HbA1c levels between baseline and follow-up CARTs, which were similar to findings from previous studies [24–26]. Patients with rapid CAN progression had higher glycemic and LDL-cholesterol levels than other patients, although other factors, including age, diabetes duration, presence of hypertension, BMI, and nephropathy, were not different between the patient groups. Poor glycemic control plays a central role in CAN progression, and intensive glycemic control and lipid modification can slow or delay the progression [27]. Hyperglycemia and dyslipidemia cause several metabolic pathways to enter a vicious cycle, resulting in the accumulation of toxic metabolic derivatives that contribute to neuronal damage and the development of micro- and macrovascular complications in type 2 diabetes [3, 28].

### Association between CAN progression and CVD

CAN is a predictor of CVD and mortality in patients with type 2 diabetes. The Detection of Ischemia in Asymptomatic Diabetic Subjects study reported that CAN based on an abnormal Valsalva ratio test result was strongly associated with silent ischemia, independent of traditional CVD risk factors [14]. The Action to Control Cardiovascular Risk in Diabetes trial also showed that the presence of CAN was a significant predictive factor of cardiovascular mortality after adjustment for traditional CV risk factors [10]. We previously reported a significant association between CAN and ischemic stroke in a study of 1458 type 2 diabetes patients with a 7-year follow-up and between CAN and recurrent CVD in 206 type 2 diabetes patients with a 9-year follow-up [13, 29]. Progressive stages of CAN are commonly associated with increasingly worse prognosis [6, 7]. However, there is limited evidence regarding the effect of CAN progression on CVD event. In this study, CAN progression was a significant predictor of CVD, and rapid CAN progression stage (normal to definite within 3 years) showed the highest risk for CVD development.

Several explanations are possible for the higher CVD risk in patients with CAN progression. Individuals with CAN have impaired exercise tolerance and may resume exercise because of deteriorating cardiac pain perception during increasing myocardial ischemia [30]. The initial development of CAN in patients with diabetes is characterized by the relative augmentation of cardiac sympathetic activity due to parasympathetic denervation, which can be measured with parameters utilized in this study [31]. Increased sympathetic activity may increase cardiac loading, finally leading to impaired heart function. Sympathetic augmentation associated with CAN increases catecholamine levels, which causes a cytotoxic effect on the heart and contributes to myocardial damage associated with increased mitochondrial reactive oxygen species and apoptosis [32–34]. CAN also directly causes diastolic filling dysfunction and reduces left ventricle ejection fraction that can contribute to the development of cardiomyopathy and cardiac dysfunction, subsequently leading to an increased risk of CVD and mortality [35, 36]. In contrast, fewer studies have assessed the association between CAN and cerebrovascular disease. Autonomic imbalance may alter cerebral regulation and variability in cerebral blood flow regulation, leading to overt cerebrovascular events [37]. In our study, the risk of CVD in the group with rapid CAN progression was higher than that in the group with consistently maintained definite CAN from baseline to follow-up CARTs. Rapid CAN progression might have more detrimental effects on the cardiovascular system, resulting in poorer outcomes [1, 6]. In addition, patients with definite CAN at baseline CARTs received more insulin, blood pressure medications, and statin treatment than those with normal or early CAN.

There are other possible candidates, including glycemic variability, that can explain the interrelation mechanisms among metabolic disorder, rapid CAN progression, and CVD. Glycemic variability triggers oxidative stress as sustained hyperglycemia and affects the CVD event in association with insulin resistance or cellular metabolic memory [20, 38]. CAN progression may also affect glycemic variability because the pancreatic β-cell is heavily innervated by parasympathetic fibers that stimulate β cells to release insulin. However, in our study, standard deviation in HbA1c levels did not have a significant difference in predicting CVD. Further studies are needed to clarify this interrelationship.

### Effect modification for the association between CAN and CVD

In this study, the magnitude of the risk of developing CVD was much higher in younger aged individuals, those with a shorter diabetes duration, or those with a lower BMI than in older patients, those with a longer diabetes duration, or those with a higher BMI. The former patients had a lower risk of CVD. In these patients, CAN progression may have a much greater impact on CVD development than other traditional CVD risk factors. In contrast, although poor glycemic control patients already have more risk factors for CVD, the effect of CAN progression on CVD was enhanced in patients with a poor glycemic status. This result demonstrated that poor glycemic control can have additional augmented effects on the association between CAN progression and CVD. The effect modification of dyslipidemia on the association between CAN progression and CVD was not shown in this study. However, it is difficult to explain the detailed mechanism of the effect modifications, and further analysis is required to clarify the issue.

### Limitations and strengths of this study

This study had the following limitations. First, we mainly focused on the parasympathetic function of CARTs. However, these tests, which included heart rate response to deep breathing, Valsalva maneuver, and postural change, are widely recommended because of their high reliability and reproducibility [8]. We also assessed BP response to postural change. However, only 1.9% of enrolled patients progressed to severe CAN (presence of orthostatic hypotension), and we could not determine any significant results using this patient group. Second, the number of outcomes was small (9.8% of total patients), and we could not perform subgroup analysis for coronary artery disease or cerebrovascular disease outcomes. Third, we did not assess baseline heart function or inflammatory biomarkers, which could affect the association between CAN and CVD in this study. Fourth, this study was conducted in one Asian ethnic group and one hospital-based cohort. More validation studies are needed to generalize the main hypothesis of this study. However, to the best of our knowledge, this is the first long-term, follow-up study that evaluated the association between CAN progression and CVD. We used mean HbA1c and LDL-cholesterol levels between baseline and follow-up CARTs to represent the more exact baseline glycemic and lipid status of patients.

## Conclusion

In summary, we suggest that CAN progression is an independent prognostic factor for CVD. Moreover, in our study, type 2 diabetes patients with rapid CAN progression had the greatest risk of CVD development. Rapid CAN progression was associated with poor glycemic control and poor LDL-cholesterol levels and regular screening for CAN is important to determine CVD risk. However, additional studies are required to clarify the precise mechanisms underlying the association between CAN progression and CVD and to apply these findings to other cohort or ethnic groups.

## Additional files

**Additional file 1: Figure S1.** Study design summarization of the sample recruitment and follow-up. CART, cardiovascular autonomic reflex test; CVD, cardiovascular disease

**Additional file 2: Table S1.** Screening intervals according to the CAN status. **Table S2.** Descriptive characteristics according to the progression status of cardiovascular autonomic neuropathy including the group with definite CAN. **Table S3.** Crude and multivariable Cox proportional hazard model for cardiovascular disease and sensitive analysis for cardiovascular disease after exclusion of the patients who developed cardiovascular disease within 2 years.

## Abbreviations

ARB: angiotensin II receptor blocker
CAN: cardiovascular autonomic neuropathy
CARTs: cardiovascular autonomic reflex tests
CVD: cardiovascular disease
HDL: high-density lipoprotein
LDL: low-density lipoprotein
