# Supplementary material for: Progression of cardiovascular autonomic neuropathy and cardiovascular disease in type 2 diabetes
Source: Cardiovasc Diabetol. 2018 Aug 2;17:109. doi: 10.1186/s12933-018-0752-6 (PMC6071370; doi:10.1186/s12933-018-0752-6)
Supplement: Supplementary file 1 — Additional file 1: Figure S1. Study design summarization of the sample recruitment and follow-up. CART, cardiovascular autonomic reflex test; CVD, cardiovascular disease [file 12933_2018_752_MOESM1_ESM.docx]

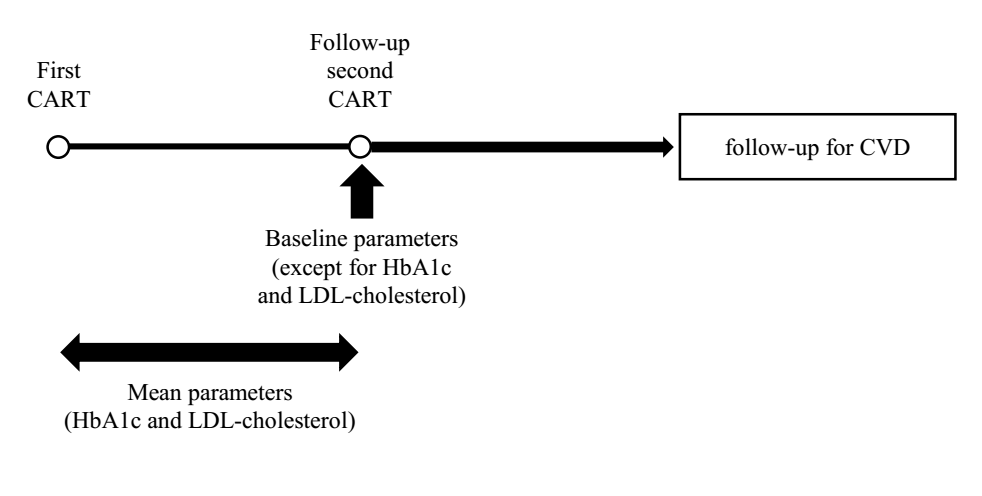


Additional file 1: Figure S1. Study design summarization of the sample recruitment and follow-up. CART, cardiovascular autonomic reflex test; CVD, cardiovascular disease.
