# Supplementary material for: Progression of cardiovascular autonomic neuropathy and cardiovascular disease in type 2 diabetes
Source: Cardiovasc Diabetol. 2018 Aug 2;17:109. doi: 10.1186/s12933-018-0752-6 (PMC6071370; doi:10.1186/s12933-018-0752-6)
Supplement: Supplementary file 2 — Additional file 2: Table S1. Screening intervals according to the CAN status. Table S2. Descriptive characteristics according to the progression status of cardiovascular autonomic neuropathy including the group with definite CAN. Table S3. Crude and multivariable Cox proportional hazard model for cardiovascular disease and sensitive analysis for cardiovascular disease after exclusion of the patients who developed cardiovascular disease within 2 years. [file 12933_2018_752_MOESM2_ESM.docx]

Additional file 2: Table S1. Screening intervals according to the CAN status

|  | CAN progression (-) | CAN progression (+) | *P*-value |
| --- | --- | --- | --- |
| Screening interval  between CARTs (years) | 2.2 ± 0.8 | 2.3 ± 0.7 | 0.126 |

|  | Non-progression | Normal → Early | Early → Definite | Normal → Definite | *P*-value |
| --- | --- | --- | --- | --- | --- |
| Screening interval  between CARTs (years) | 2.2 ± 0.8 | 2.3 ± 0.7 | 2.2 ± 0.8 | 2.6 ± 0.6 | 0.079 |

Additional file 2: Table S2. Descriptive characteristics according to the progression status of cardiovascular autonomic neuropathy including the group with definite CAN.

|  | Non-progression | Normal → Early | Early → Definite | Normal → Definite | Definite → Definite | Definite → Normal or Early | *P*-value |
| --- | --- | --- | --- | --- | --- | --- | --- |
|  | (N=404) | (N=79) | (N=65) | (N=30) | (N=42) | (N=12) |  |
| Women, n (%) | 220 (54.5) | 49 (62.0) | 41 (63.1) | 19 (63.3) | 26 (61.9) | 7 (58.3) | 0.566 |
| Age (years) | 57.1 ± 10.1 | 59.9 ± 10.0 | 62.0 ± 11.1 | 60.7 ± 8.8 | 63.7 ± 8.1 | 56.2 ± 8.5 | <0.001 |
| Diabetes duration (years) | 9.1 ± 5.7 | 11.4 ± 6.6 | 14.2 ± 7.1 | 11.5 ± 5.6 | 13.6 ± 6.9 | 10.4 ± 5.0 | <0.001 |
| Body mass index (kg/m^2^) | 24.8 ± 3.2 | 24.2 ± 3.4 | 24.7 ± 3.4 | 25.7 ± 2.9 | 25.4 ± 3.7 | 24.3 ± 3.1 | 0.319 |
| Hypertension, n (%) | 163 (42.2) | 35 (46.7) | 31 (50.0) | 13 (44.8) | 25 (59.5) | 6 (50.0) | 0.255 |
| Smoking, n (%) | 93 (23.0) | 12 (15.2) | 15 (23.1) | 4 (13.3) | 11 (26.2) | 2 (16.7) | 0.501 |
| Alcohol, n (%) | 105 (26.0) | 18 (22.8) | 13 (20.0) | 2 (6.7) | 13 (31.0) | 1 (8.3) | 0.098 |
| Insulin, n (%) | 87 (21.5) | 14 (17.7) | 30 (46.2) | 10 (33.3) | 21 (50.0) | 1 (8.3) | <0.001 |
| ACE inhibitor/ARBs, n (%) | 122 (30.2) | 25 (31.6) | 24 (36.9) | 10 (33.3) | 17 (40.5) | 3 (25.0) | 0.730 |
| Calcium channel blocker, n (%) | 63 (15.6) | 20 (25.3) | 13 (20.0) | 5 (16.7) | 12 (28.6) | 4 (33.3) | 0.106 |
| Aspirin, n (%) | 28 (6.9) | 7 (8.9) | 6 (9.2) | 4 (13.3) | 4 (9.5) | 0 (0.0) | 0.664 |
| Statin, n (%) | 44 (10.9) | 9 (11.4) | 8 (12.3) | 4 (13.3) | 7 (16.7) | 1 (8.3) | 0.911 |
| FPG (mmol/L) | 8.3 ± 2.7 | 8.4 ± 2.8 | 9.1 ± 4.3 | 9.4 ± 1.5 | 9.6 ± 3.9 | 8.4 ± 2.8 | 0.001 |
| eGFR (mL/min/1.73 m^2^) | 86.7 ± 16.1 | 84.3 ± 17.7 | 81.1 ± 20.6 | 85.2 ± 18.9 | 72.5 ± 19.5 | 81.4 ± 20.4 | 0.042 |
| Mean HbA1c (%) | 8.1 ± 1.5 | 8.2 ± 1.4 | 9.2 ± 1.6 | 9.2 ± 1.4 | 9.0 ± 1.8 | 8.6 ± 1.4 | <0.001 |
| SD HbA1c | 1.1 ± 1.0 | 0.7 ± 0.6 | 1.3 ± 1.3 | 1.2 ± 1.1 | 1.3 ± 1.1 | 1.1 ± 1.2 | 0.285 |
| Mean HbA1c (mmol/L) | 65.1 ± 16.0 | 64.8 ± 16.3 | 76.9 ± 17.3 | 77.4 ± 15.6 | 68.2 ± 29.3 | 70.9 ± 15.4 | <0.001 |
| Mean total cholesterol (mmol/L) | 4.7 ± 0.9 | 4.7 ± 0.9 | 4.7 ± 0.9 | 5.2 ± 0.8 | 4.6 ± 1.1 | 4.7 ± 0.7 | 0.349 |
| Mean triglyceride (mmol/L) | 1.7 ± 1.0 | 1.7 ± 0.8 | 1.6 ± 0.9 | 1.8 ± 1.1 | 1.9 ± 1.0 | 1.7 ± 1.8 | 0.371 |
| Mean HDL-cholesterol (mmol/L) | 1.2 ± 0.3 | 1.1 ± 0.3 | 1.1 ± 0.3 | 1.2 ± 0.3 | 1.0 ± 0.3 | 1.2 ± 0.2 | 0.062 |
| Mean LDL-cholesterol (mmol/L) | 2.7 ± 0.8 | 2.8 ± 0.8 | 2.8 ± 0.9 | 3.2 ± 0.6 | 2.7 ± 0.9 | 2.8 ± 1.0 | 0.196 |
| UAE (mg/day) | 48.1 ± 191.4 | 142.5 ± 481.0 | 182.7 ± 513.5 | 120.4 ± 137.9 | 278.8 ± 779.3 | 11.3 ± 8.1 | 0.001 |

Values are presented as number (%) or mean ± SD. CVD, cardiovascular disease; ARB, angiotensin receptor blocker; FPG, fasting plasma glucose; eGFR, estimated glomerular filtration rate; SD, standard deviation; UAE, urinary albumin excretion

Additional file 2: Table S3. Crude and multivariable Cox proportional hazard model for cardiovascular disease and sensitive analysis for cardiovascular disease after exclusion of the patients who developed cardiovascular disease within 2 years.

|  | Crude HR (95% CI) | *P* value | Age and sex adjusted HR (95% CI) | *P* value | Fully adjusted HR (95% CI) | *P* value | *P* for interaction |
| --- | --- | --- | --- | --- | --- | --- | --- |
| **Subgroup analysis by CAN status** | | | | | | | |
| Non-progression | Reference |  | Reference |  | Reference |  |  |
| Normal to early | 2.73 (1.28-5.81) | 0.009 | 2.23 (1.04-4.78) | 0.038 | 2.17 (0.99-4.74) | 0.052 |  |
| Early to definite | 5.18 (2.63-10.20) | <0.001 | 4.15 (2.06-8.36) | <0.001 | 3.30 (1.57-6.92) | 0.002 |  |
| Normal to definite | 6.99 (3.19-15.32) | <0.001 | 6.43 (2.93-14.09) | <0.001 | 5.41 (2.38-12.31) | <0.001 |  |
| Definite to definite | 3.98 (1.85-8.53) | <0.001 | 2.93 (1.35-6.38) | 0.007 | 2.42 (1.06-5.51) | 0.036 |  |
| Definite to normal or early | 1.98 (0.45-8.78) | 0.368 | 2.30 (0.52-10.25) | 0.274 | 2.26 (0.49-10.36) | 0.293 |  |

CVD, cardiovascular disease; CAN, cardiovascular autonomic neuropathy
